# Preparative Isolation and Purification of Three Sesquiterpenoid Lactones from *Eupatorium lindleyanum* DC. by High-Speed Counter-Current Chromatography

**DOI:** 10.3390/molecules17089002

**Published:** 2012-07-27

**Authors:** Guilong Yan, Lilian Ji, Yuming Luo, Yonghong Hu

**Affiliations:** 1 College of Biotechnology and Pharmaceutical Engineering, Nanjing University of Technology, Nanjing 210009, China; 2 Jiangsu Key Laboratory for Eco-Agricultural Biotechnology around Hongze Lake, Huaiyin Normal University, Huaian 223300, China; 3 Jiangsu Key Laboratory for Biomass-based Energy and Enzyme Technology, Huaiyin Normal University, Huaian 223300, China

**Keywords:** *Eupatorium lindleyanum* DC., high-speed counter-current chromatography, sesquiterpenoid lactone, separation

## Abstract

A high-speed counter-current chromatography (HSCCC) method was established for the preparative separation of three sesquiterpenoid lactones from *Eupatorium lindleyanum* DC. The two-phase solvent system composed of *n*-hexane–ethyl acetate–methanol–water (1:4:2:3, v/v/v/v) was selected. From 540 mg of the *n*-butanol fraction of *Eupatorium lindleyanum* DC., 10.8 mg of 3β-hydroxy-8β-[4'-hydroxy-tigloyloxy]-costunolide, 17.9 mg of eupalinolide A and 19.3 mg of eupalinolide B were obtained in a one-step HSCCC separation, with purities of 91.8%, 97.9% and 97.1%, respectively, as determined by HPLC. Their structures were further identified by ESI-MS and ^1^H-NMR.

## 1. Introduction

*Eupatorium lindleyanum* DC. (family Compositae) is a traditional Chinese herb. Its aerial part, called ‘Yemazhui’, shows antihistaminic, antibacterial and antioxidant activities [1,2,3], and is traditionally used for the treatment of inﬂammation and asthma, as well as throat disorders [1]. Previous phytochemical investigations [4,5,6,7] on this species have been found a variety of secondary metabolites such as isoﬂavonoids, sesquiterpenoid lactones, and triterpeniods. Among them, the sesquiterpenoid lactones, which had very similar skeletons and considerable diversity in number, were considered as the major active constituents [6], so the development of rapid and economical methods for the identification of these sesquiterpenoid lactones is of great significance. However, their preparative separation and purification by conventional methods is tedious, time consuming, with low recoveries and high cost [8,9].

**Figure 1:**
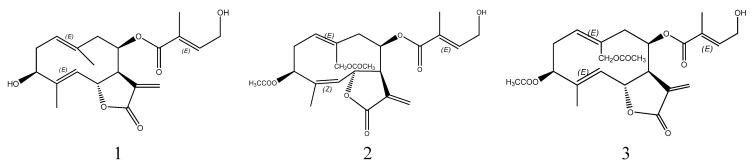
Chemical structures of three sesquiterpenoid lactones from *Eupatorium lindleyanum* DC. (1: 3β-Hydroxy-8β-[4′-hydroxytigloyloxy]-costunolide; 2: eupalinolide A; 3: eupalinolide B)

High-speed counter-current chromatography (HSCCC) is a liquid-liquid chromatographic technique based on solvent partition that eliminates irreversible adsorption of samples onto the solid separation materials and produces excellent sample recoveries [10]. Thus, this method has been widely used for separation and purification of various natural products [9,11,12,13,14,15,16]. However, there is no report on the use of HSCCC for the isolation and purification of sesquiterpenoid lactones from *Eupatorium lindleyanum* DC, thus we report herein an efficient method for the preparative isolation and purification of three sesquiterpenoid lactones (structures shown in Figure 1) from *Eupatorium lindleyanum* DC. by one-step HSCCC.

## 2. Results and Discussion

### 2.1. Selection of Two-Phase Solvent System and Other Separation Conditions of HSCCC

In an HSCCC separation, the selection of a suitable two-phase solvent system is the first and most important step, and a good solvent system can provide an ideal partition coefficient (*K*) for the target compounds in the range of 0.5–2.0. In this experiment, the solvent system composed of n-hexane-ethyl acetate-methanol-water at different volume ratios was investigated and the *K* values of the target compounds were shown in Table 1.

The results indicated that the solvent system of *n*-hexane–ethyl acetate–methanol–water at the volume ratios of 2:4:3:2 (v/v/v/v) had small *K* values for the three compounds, and the solvent system *n*-hexane–ethyl acetate–methanol–water (1:4:3:3, v/v/v/v) provided suitable *K* values for separating compounds 2 (*K* value: 0.61) and 3 (*K* value: 1.26) but was unsuitable for compound 1 (*K* value: 0.26). On the other hand, the *K* values of compound 2 (*K* value: 3.38) and 3(*K* value: 5.51) were very high in the solvent system *n*-hexane–ethyl acetate–methanol–water (1:8:4:6, v/v/v/v), but when *n*-hexane–ethyl acetate–methanol–water (1:4:2:3, v/v/v/v) was used as the two phase solvent system, the *K* values of all the compounds were suitable for separation. Therefore, this two-phase solvent system was selected for the HSCCC separation in the present paper.

**Table 1:** The partition coefficient (*K*) values of the three sesquiterpenoid lactones in different solvent systems.

*n*-hexane-ethyl acetate-methanol-water	Partition coefficient (*K*)		
(v/v/v/v)	*K_1_*	*K_2_*	*K_3_*
2:4:3:2	0.06	0.14	0.28
1:4:3:3	0.26	0.61	1.26
1:4:2:3	0.54	1.40	1.94
1:8:4:6	1.64	3.38	5.51

Other factors, such as the revolution speed of the separation column and the flow rate of the mobile phase, were also investigated. The results showed that when the flow rate was 2.0 mL/min and the revolution speed was 900 rpm, the retention percentage of the stationary phase could reach 50% and good separation results could be achieved.

### 2.2. HSCCC Separation of Crude Sample

**Figure 2:**
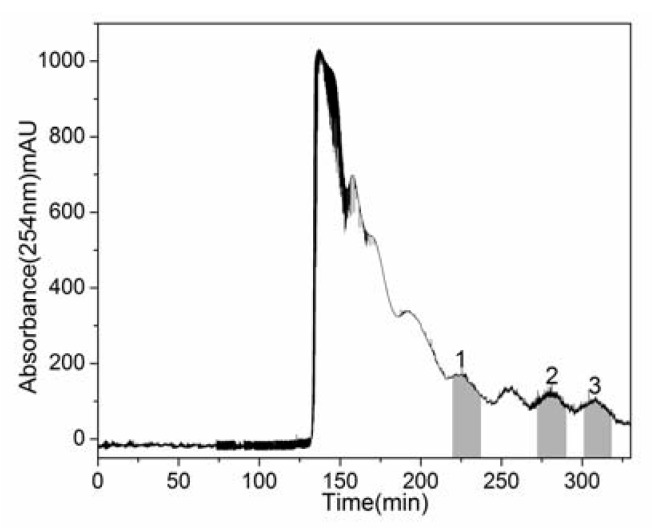
HSCCC separation chromatogram of *n*-butanol fraction of the *Eupatorium lindleyanum* DC. ethanol extract. Solvent system: *n*-hexane-ethyl acetate-methanol-water (1:4:2:3, v/v/v/v); flow rate: 2.0 mL/min; revolution speed: 900 rpm; detection wavelength: 254 nm; column temperature: 25 °C; sample: 540 mg of *n*-butanol fraction dissolved in 10 mL two-phase solvent system. Peaks 1, 2 and 3 correspond to 3β-hydroxy-8β-[4'-hydroxytigloyloxy]-costunolide, eupalinolide A and eupalinolide B, respectively.

Under the optimum separation conditions, about 540 mg of *n*-butanol fraction was separated and purified in one step by HSCCC. The corresponding HSCCC chromatogram is shown in Figure 2.

**Figure 3:**
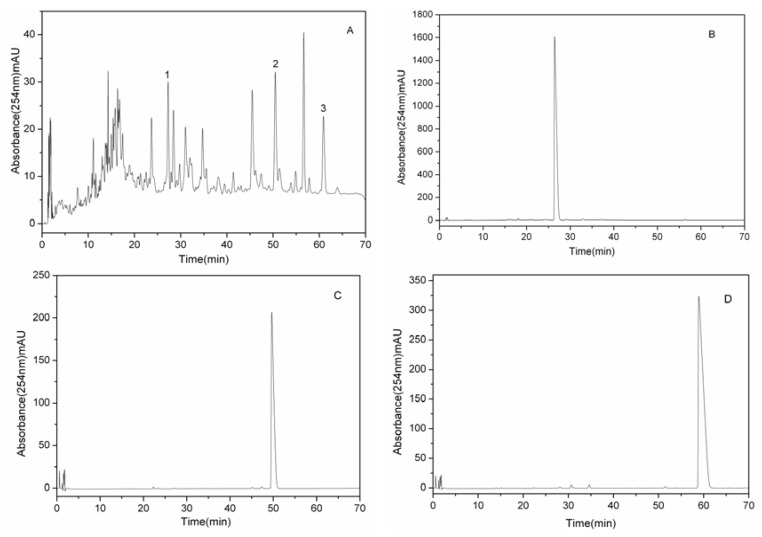
HPLC chromatograms of n-butanol fraction of the *Eupatorium lindleyanum* DC. ethanol extract and HSCCC peak fractions. (**A**) Crude sample; (**B**) Peak 1 in Figure 2; (**C**) Peak 2 in Figure 2; (**D**) Peak 3 in Figure 2.

The separation time was about 330 min in each separation run. Figure 2 showed that the three compounds were eluted in order of increasing *K* value and good resolution could be obtained. HSCCC is based on the principle of the partition coefficient of the solutes between the stationary and mobile phases [10], so the solutes were eluted from the multilayer coil separation columns in order of their partition coefficient. Finally three fractions were collected, the fraction from peak 1 of this separation yielded 10.8 mg of compound **1**, the fraction from peak 2 yielded 17.9 mg of compound **2**, and the fraction from peak 3 yielded 19.3 mg of compound **3**. The purities of compounds **1**–**3** were 91.8%, 97.9% and 97.1%, respectively, as determined by HPLC (Figure 3B–D).

## 3. Experimental

### 3.1. Reagents and Materials

Analytical-grade reagents used for HSCCC separation were purchased from Sinopharm Chemical Reagent Co., Ltd (Shanghai, China). HPLC grade acetonitrile was purchased from the Jiangsu Hanbon Science & Technology Co., Ltd. (Huaian, China). All aqueous solutions were prepared with pure water produced by direct-Q3 system (Millipore, Billerica, MA, USA).

### 3.2. Apparatus

The HSCCC instrument used in the present study was a TBE-300B high-speed counter-current chromatography system (Shanghai Tauto Biotech Co., Ltd., Shanghai, China) with three multilayer coil separation columns connected in series (total volume: 280 mL, internal diameter of tubing: 1.6 mm) and a 20 mL sample loop. The revolution radius was 5 cm, and the β-values of the multilayer coil varied from 0.5 at internal to 0.8 at the external terminal (β = r/R, where r is the distance from the coil to the holder shaft, and R is the revolution radius or the distance between the holder axis and central axis of the centrifuge). The revolution speed of the apparatus was adjustable from 0 rpm to 1,000 rpm. An HX 105 constant-temperature circulating implement (Beijing Changliu Scientific Instrument, Beijing, China) was used to control the separation temperature. The system was also equipped with a TBP5002 constant flow pump (Shanghai Tauto Biotech Co., Ltd., Shanghai, China), a model Quik Sep UV-50 UV-vis monitor (H&E Co., Ltd, Beijing, China). HIE-50A chromatography workstation (H&E Co., Ltd, Beijing, China) was employed to record the chromatogram. The HPLC equipment used was an Agilent 1260 system including G1311C Quat Pump, G1314B VWD, G1329B ALS, G1316A TCC and Agilent ChemStation workstation (Agilent Technologies, Waldbronn, Germany). ESI-MS spectrums were obtained with a Thermo LCQ advantage ion-trap mass spectrometer (Thermo, San Jose, CA, USA). The nuclear magnetic resonance (NMR) spectrometer was a Bruker Avance 400 NMR system (Bruker, Fallanden, Switzerland).

### 3.3. Plant Material

Plant material (*Eupatorium lindleyanum* DC. 30 kg) was purchased from Xuyi County of Jiangsu Province, China, and identified by one of the authors (Yuming Luo). The voucher specimens were deposited at the laboratory of the School of Life Sciences, Huaiyin Normal University.

### 3.4. Preparation of Samples

The extract from *Eupatorium lindleyanum* DC. was prepared bythe method mentioned in reference [3]. The dried, powdered aerial parts of *E. lindleyanum* DC (10.0 kg) was extracted three times with 95% EtOH (100 L × 3 d) at ambient temperature. After removing the solvent under reduced pressure, the ethanol extract was suspended in water and then partitioned with petroleum ether, ethyl acetate and *n*-butanol. The final yield of *n*-butanol fraction (BF) from the *E. lindleyanum* DC. ethanol extract was 68.21 g. A portion of this fraction sample was subjected to HSCCC for further isolation and separation.

### 3.5. Selection of Two-Phase Solvent System

The two-phase composition was determined by the partition coefficient (*K* values), which were determined by HPLC as follows: About 6 mg of sample was placed in a 5 mL test tube, to which 1 mL of each phase of the equilibrated two-phase solvent system was added. After thorough mixing and settling, 0.5 mL of each layer was taken out and evaporated to dryness separately. The residues were diluted with methanol to 1 mL and analyzed by HPLC. The *K* values were defined as the peak area of target compounds in the upper phase divided by their peak area in the lower phase.

### 3.6. Preparation of Two-Phase Solvent System and Sample Solution

The selected two-phase solvent system was prepared by adding the solvent to a separation funnel according to the volume ratios and thoroughly equilibrated by repeated vigorous shaking at room temperature. The two phases were separated and degassed by sonication for 30 min shortly before use. The sample solution was prepared by dissolving the *n*-butanol fraction from *E. lindleyanum* DC. in the mixture solution of 5 mL upper phase and 5 mL lower phase of the solvent system.

### 3.7. HSCCC Separation Procedure

In each HSCCC separation, the multiplayer coiled column was initially filled with the upper phase (stationary phase). The apparatus was then rotated at 900 rpm, and the lower phase (mobile phase) was pumped into the column at a flow rate of 2.0 mL/min in the head-to-tail elution mode. After the mobile phase front emerged and hydrodynamic equilibrium was established, about 10 mL sample solution containing 540 mg *n*-butanol fraction was injected through the sample injection valve. The effluent from the outlet was continuously monitored with a UV-vis detector at 254 nm. The temperature of the apparatus was set at 25 °C. Peak fractions were collected manually according to chromatographic peak profiles.

### 3.8. Analyses and Identification of HSCCC Peak Fractions

The crude sample and each purified fractions isolated from the HSCCC were analyzed by HPLC on a Zorbax Eclipse XDB-C18 column (100 mm, 4.6 mm id, 3μm, Agilent) with the column temperature at 30 °C. The solvent system was a mixture of water (solvent A) and acetonitrile (solvent B), and the solvent gradient was as follows: 0–10 min from 90% solvent A to 80% solvent A, 10–15 min isocratic 80% solvent A, 15–65 min from 80% solvent A to 68% solvent A, 65–70 min from 68% solvent A to 90% solvent A. The flow rate was 1.0 mL/min and the effluent was monitored at 254 nm by a UV detector. The identification of HSCCC peak fractions was carried out by ESI-MS and ^1^H-NMR spectroscopy.

### 3.9. Structural Identification

The structural identification of the fractions was performed according to ESI-MS and ^1^H-NMR as follows:

Peak 1: ESI-MS: *m/z* 421.7 [M + CH3COOH]^−^; ^1^H-NMR (400 MHz, CDCl_3_): 4.93 (1H, br dd, 11.8, 3.1, H-1); 2.51 (1H, m, H-2a); 2.34 (1H, m, H-2b); 4.34 (1H, signal overlapped, H-3); 4.85 (1H, br d, 9.9, H-5); 5.21 (1H, t like, 9.4, 9.2, H-6); 2.92 (1H, m, H-7); 5.80 (1H, br d, 2.7, H-8); 2.84 (1H, dd, 14.4, 4.5, H-9a); 2.30 (1H, signal overlapped, H-9b); 6.32 (1H, d, 3.3, H-13a); 5.63 (1H, d, 2.8, H-13b); 1.52 (3H, br s, H-14); 1.81 (3H, br s, H-15); 6.79 (1H, m, H-3′); 4.38 (2H, signal overlapped, H-4′); 1.84 (3H, br s, H-5′). Comparing the above data with the literature data [17], compound **1** was identified as 3β-Hydroxy-8β-[4′-hydroxytigloyloxy]-costunolide.

Peak 2: ESI-MS: *m/z* 463.3 [M + H]^+^; ^1^H-NMR (400 MHz, CDCl_3_): 5.27 (1H, signal overlapped, H-1); 2.43 (1H, m, H-2a); 2.75 (1H, m, H-2b); 5.30 (1H, signal overlapped, H-3); 5.23 (1H, br d, 10.9, H-5); 5.83 (1H, dd, 11.2, 9.9, H-6); 2.98 (1H, m, H-7); 5.49 (1H, m, H-8); 2.30 (1H, br d, 14.2, H-9a); 3.15 (1H, br d, 13.4, H-9b); 6.38 (1H, d, 2.0, H-13a); 5.80 (1H, d, 2.0, H-13b); 4.90 (1H, d, 13.0, H-14a); 4.71 (1H, d, 13.0, H-14b); 1.84 (3H, br s, H-15); 6.76 (1H, t, 5.3, H-3′); 4.30 (2H, m, H-4′); 1.80 (3H, br s, H-5′); 2.01 (3H, s, CH_3_COO); 2.19 (3H, s, CH_3_COO). Comparing the above data with the literature data [18], compound **2** was identified as eupalinolide A.

Peak 3: ESI-MS: m/z 463.4 [M + H]^+^;^1^H-NMR (400MHz, CDCl_3_): 5.37 (1H, signal overlapped, H-1); 2.32 (1H, m, H-2a); 3.04 (1H, signal overlapped, H-2b); 5.26 (1H, signal overlapped, H-3); 5.36 (1H, signal overlapped, H-5); 5.24 (1H, signal overlapped, H-6); 3.00 (1H, m, H-7); 5.51 (1H, m, H-8); 2.21 (1H, m, H-9a); 2.89 (1H, m, H-9b); 6.38 (1H, d, 2.1, H-13a); 5.80 (1H, d, 2.0, H-13b); 4.97 (1H, d, 12.6, H-14a); 4.70 (1H, d, 12.7, H-14b); 1.81 (3H, br s, H-15); 6.77 (1H, t, 5.6, H-3′); 4.30 (2H, d, 5.9, H-4′); 1.81 (3H, br s, H-5′); 2.03 (3H, s, CH_3_COO); 2.11 (3H, s, CH_3_COO). The ^1^H spectral data were very similar to that of compound **2**, except that H-6 showed a significant down-field shift. Comparing the above data with the literature data [18], compound **3** was identified as eupalinolide B.

## 4. Conclusions

As is known the separation of natural compounds with similar structures is very difficult. In the present study, three similar sesquiterpenoid lactones were successfully isolated for the first time from *Eupatorium lindleyanum* DC. in high purity by conventional HSCCC. The results of our study demonstrate that HSCCC is an effective method for separating some difficult to separate natural compounds.

## Supplementary Materials

Supplementary materials can be accessed at: http://www.mdpi.com/1420-3049/17/8/9002/s1.
