# Supplementary material for: Preparative Isolation and Purification of Three Sesquiterpenoid Lactones from Eupatorium lindleyanum DC. by High-Speed Counter-Current Chromatography
Source: Molecules. 2012 Jul 27;17(8):9002–9. doi: 10.3390/molecules17089002 (PMC6268613; doi:10.3390/molecules17089002)

^{1}H-NMR spectroscopy for 3β-Hydroxy-8β-[4'-hydroxytigloyloxy]-costunolide.

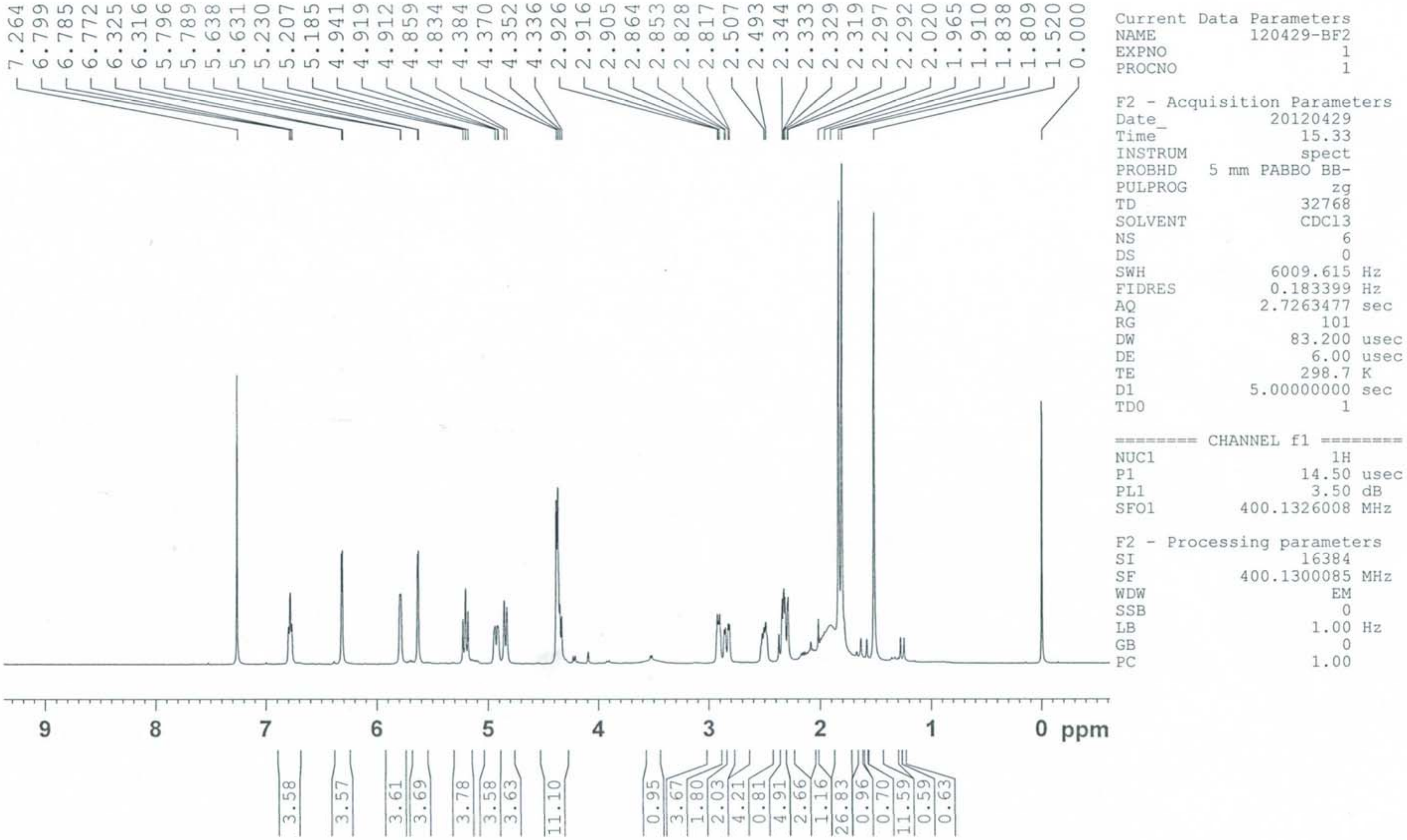

^{1}H-NMR spectroscopy for eupalinolide A.

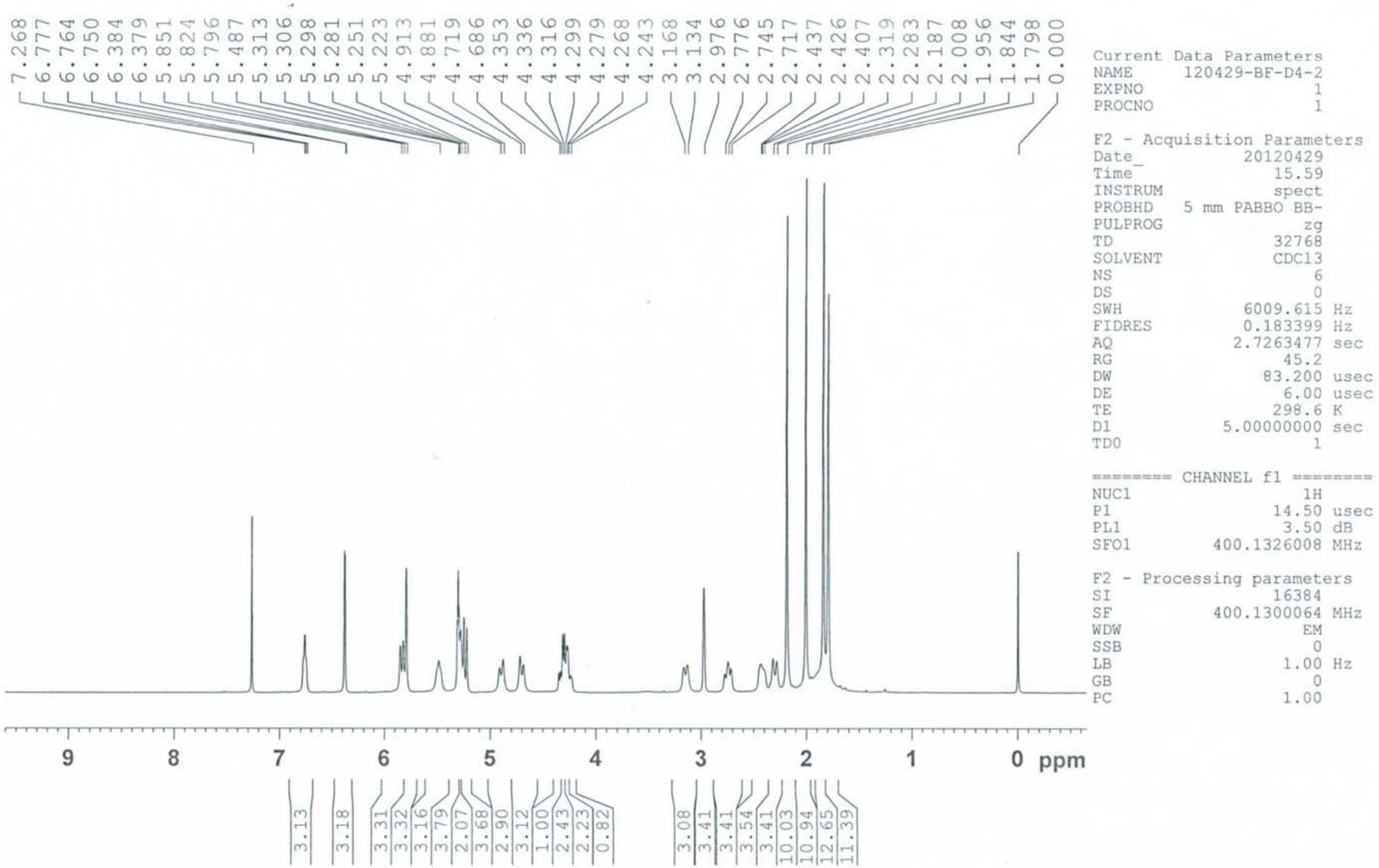

^{1}H-NMR spectroscopy for eupalinolide B.

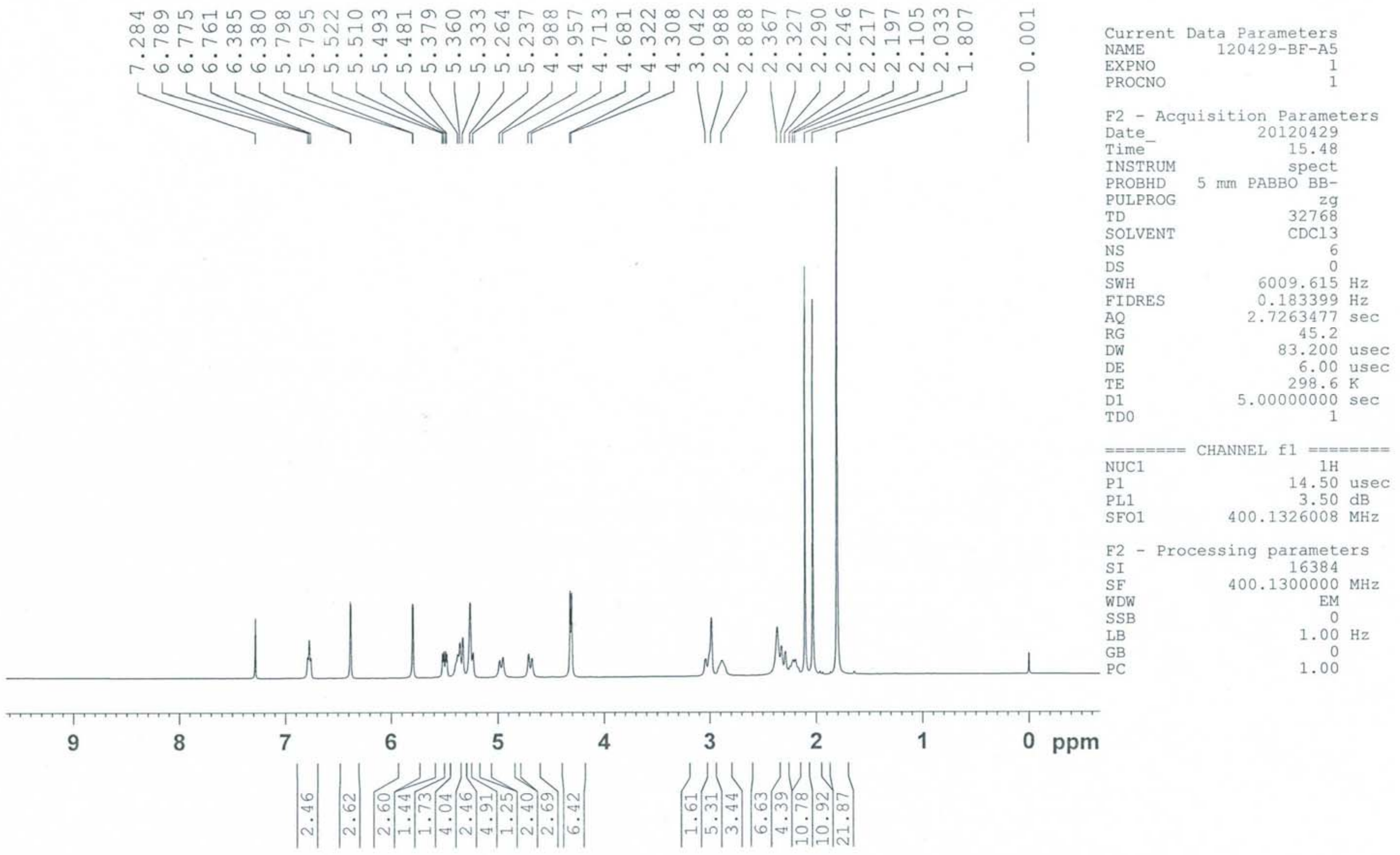

Mass spectrogram for 3β-Hydroxy-8β-[4'-hydroxytigloyloxy]-costunolide.

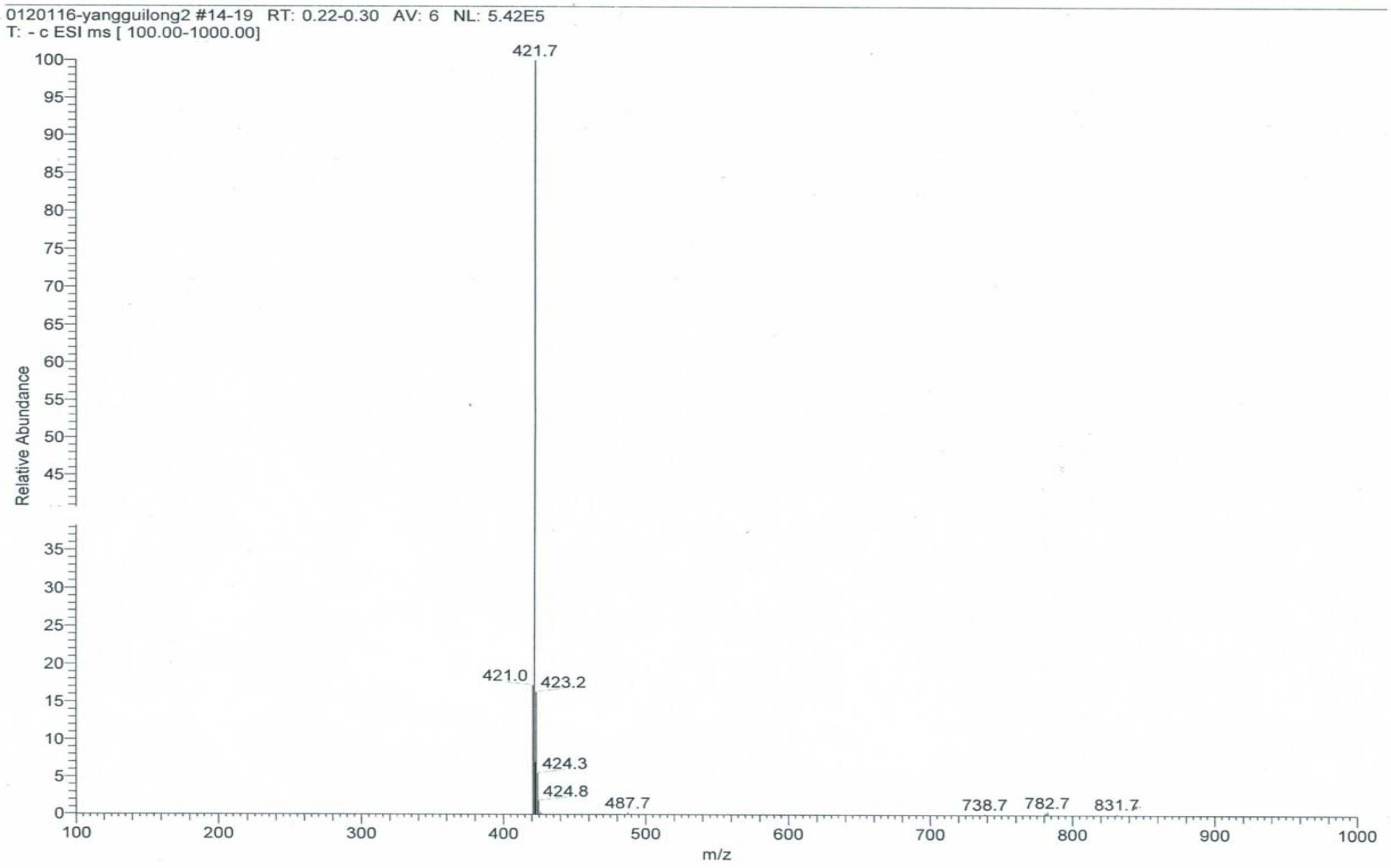

Mass spectrogram for eupalinolide A.

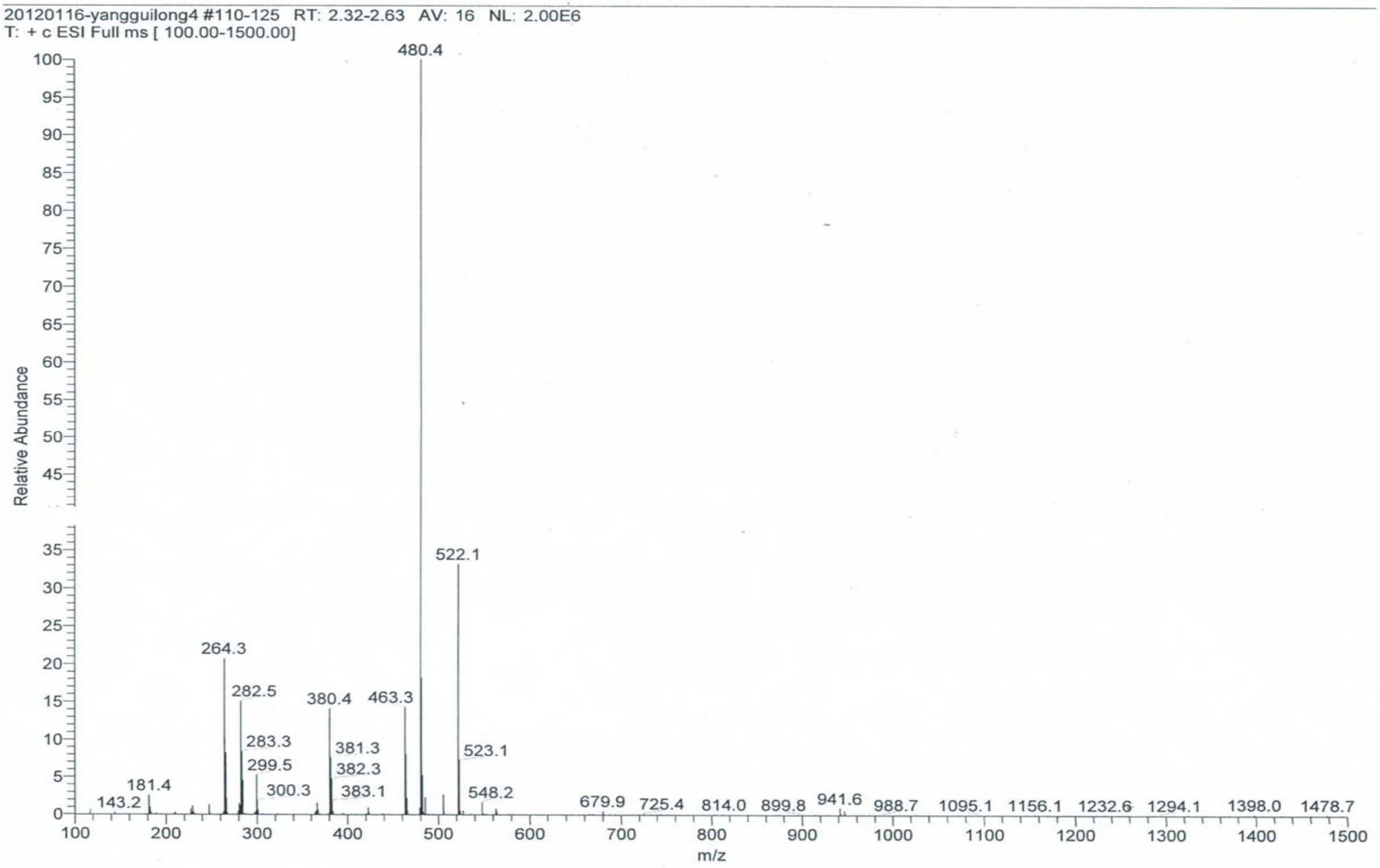

Mass spectrogram for eupalinolide B.

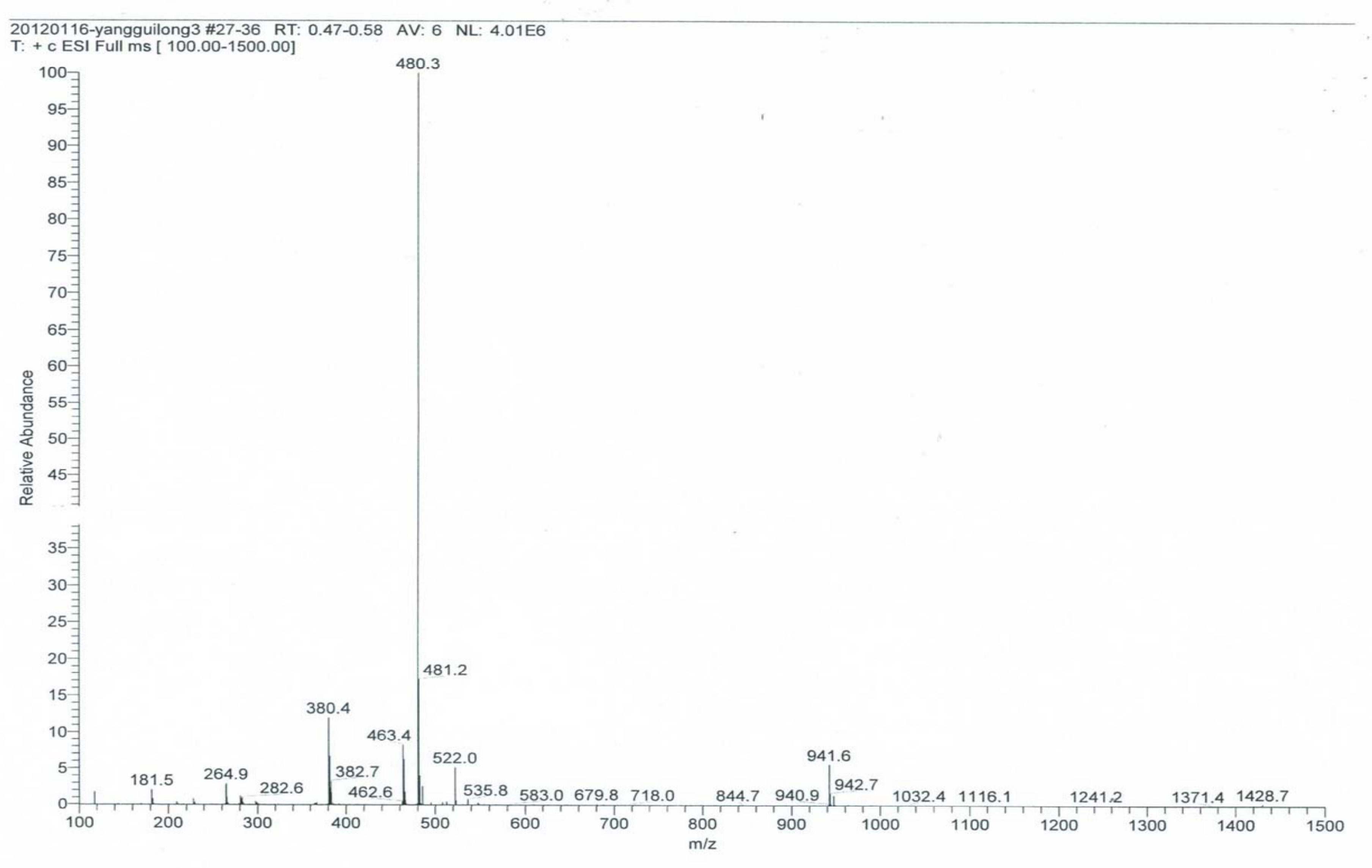

Supplement: Supplementary file 1 [file molecules-17-09002-s001.pdf]
